# Cardiovascular risk reduction with once-weekly semaglutide in subjects with type 2 diabetes: a post hoc analysis of gender, age, and baseline CV risk profile in the SUSTAIN 6 trial

**DOI:** 10.1186/s12933-019-0871-8

**Published:** 2019-06-06

**Authors:** Lawrence A. Leiter, Stephen C. Bain, Irene Hramiak, Esteban Jódar, Sten Madsbad, Theis Gondolf, Thomas Hansen, Ingrid Holst, Ildiko Lingvay

**Affiliations:** 1 Division of Endocrinology and Metabolism, Li Ka Shing Knowledge Institute, St. Michael’s Hospital, University of Toronto, 61 Queen St. East #6121, Toronto, ON M5C 2T2 Canada; 2 Diabetes Research Unit Cymru, Swansea University Medical School, Swansea, Wales UK; 3 University of Western Ontario, London, ON Canada; 4 Hospital Universitario Quirónsalud, Madrid, Spain; 5 University of Copenhagen, Hvidovre, Denmark; 6 Novo Nordisk A/S, Søborg, Denmark; 7 UT Southwestern Medical Center, Dallas, TX USA

**Keywords:** Semaglutide, Cardiovascular events, Gender, Age, Baseline cardiovascular risk, Type 2 diabetes, SUSTAIN 6, Cardiovascular outcome trial

## Abstract

**Background:**

The SUSTAIN 6 trial demonstrated that once-weekly semaglutide (0.5 and 1.0 mg) significantly reduced major adverse cardiovascular (CV) events (MACE) vs placebo in subjects with type 2 diabetes (T2D) and high CV risk. The effects of gender, age and baseline CV risk on outcomes are important considerations for further study.

**Methods:**

Subjects were grouped according to gender, age (50–65 years and > 65 years), and CV risk profile at baseline (prior myocardial infarction [MI] or stroke vs no prior MI or stroke, and established CV disease [CVD] vs CV risk factors alone, including subjects with chronic kidney disease). Time to MACE and its individual components (CV death, nonfatal MI, nonfatal stroke), hospitalization for unstable angina or heart failure, and revascularization (coronary and peripheral) were analyzed for all subgroups. Additional analyses were performed for gender and age to investigate change from baseline in HbA_1c_ and body weight, as well as tolerability.

**Results:**

A total of 3297 subjects were included. The majority of subjects (60.7%) were male; 43% were > 65 years of age; 41.5% had a history of MI or stroke; and 76.8% had established CVD. Compared with placebo, semaglutide reduced the risk of the first occurrence of MACE and each MACE component consistently across all subgroups (gender, age, and baseline CV risk profile). Revascularizations, HbA_1c_ and body weight were also reduced consistently across all subgroups compared with placebo. Gastrointestinal adverse events in all treatment groups were more common among women than men, but rates of premature treatment discontinuation were similar for both genders.

**Conclusions:**

In this post hoc analysis of SUSTAIN 6, once-weekly semaglutide vs placebo reduced the risk of MACE in all subjects included in the trial, regardless of gender, age, or baseline CV risk profile.

*Trial registry* Clinicaltrials.gov, Identifying number: NCT01720446, Date of registration: October 29, 2012
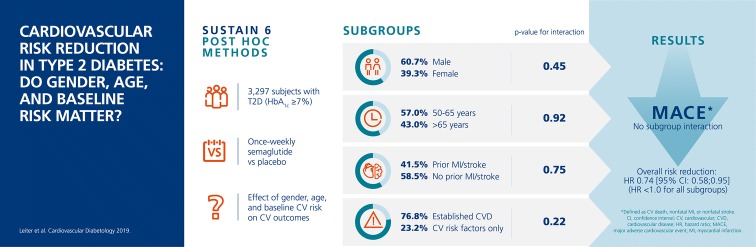

**Electronic supplementary material:**

The online version of this article (10.1186/s12933-019-0871-8) contains supplementary material, which is available to authorized users.

## Background

Cardiovascular disease (CVD) is the leading cause of morbidity and mortality in people with type 2 diabetes (T2D) [1, 2], and diabetes itself confers a substantial independent risk of coronary heart disease, stroke, and death from other vascular causes [3]. Current diabetes guidelines recommend multifactorial CV risk management and the preferential use of a glucagon-like peptide-1 receptor agonist (GLP-1RA) or sodium–glucose cotransporter-2 inhibitor with proven CV benefits as a first choice add-on to metformin in patients with T2D and established atherosclerotic CVD [2, 4]. Semaglutide is a GLP-1 analogue approved as a once-weekly, subcutaneous treatment for T2D [5, 6]. The phase 3 SUSTAIN (Semaglutide Unabated Sustainability in Treatment of Type 2 Diabetes) clinical trial program evaluated the efficacy and safety of semaglutide in subjects with T2D in a range of patient populations across the continuum of diabetes care [7–14]. In the SUSTAIN 6 CV outcomes trial (CVOT), once-weekly semaglutide (0.5 or 1.0 mg) added to standard of care significantly reduced the occurrence of a first major adverse CV event (MACE: CV death, nonfatal myocardial infarction [MI], or nonfatal stroke) vs placebo over 2 years in 3297 subjects with T2D and high CV risk [12]. Given the increasing emphasis on individualized patient care in the management of T2D [4], this post hoc analysis assessed the effects of gender, age, and baseline CV risk on the reduction of CV risk in the SUSTAIN 6 trial.

## Methods

### SUSTAIN 6 study design

The design of SUSTAIN 6 (clinicaltrials.gov NCT01720446) has been described previously [12]. In brief, SUSTAIN 6 was a randomized, double-blind, placebo-controlled, parallel-group trial to evaluate once-weekly semaglutide 0.5 or 1.0 mg vs volume-matched placebo over a 104-week treatment period plus a 5-week follow-up period. The trial was conducted in compliance with the International Conference on Harmonisation Good Clinical Practice guidelines and the Declaration of Helsinki [15, 16]. The protocol was approved by local ethics committees and institutional review boards. Written informed consent was obtained from all subjects before trial commencement.

A total of 3297 subjects with T2D (HbA_1c_ ≥ 7%) were randomized to receive once-weekly semaglutide 0.5 or 1.0 mg or placebo for 104 weeks. Subjects were ≥ 50 years of age with established CVD (defined as previous CV, cerebrovascular, or peripheral vascular disease), chronic heart failure (New York Heart Association class II or III), or chronic kidney disease (CKD) of stage 3 or higher, or were ≥ 60 years of age with at least one CV risk factor (microalbuminuria or proteinuria, hypertension and left ventricular hypertrophy, left ventricular systolic or diastolic dysfunction or ankle–brachial index < 0.9). All subjects treated with semaglutide followed a fixed dose-escalation regimen, with a starting dose of 0.25 mg for 4 weeks that escalated to 0.5 mg for 4 weeks until the maintenance dose (0.5 or 1.0 mg) was reached.

The primary composite outcome (MACE) was the first occurrence of death from CV causes, nonfatal MI, or nonfatal stroke. Other outcomes included time to first hospitalization for unstable angina and heart failure, time to first revascularization (coronary or peripheral), and changes in HbA_1c_ and weight. All outcomes were collected after 104 weeks of treatment.

### Statistical analysis

This post hoc analysis examined the effect of gender, age (subjects aged 50–65 and > 65 years), and CV risk profile at baseline on time to first occurrence of MACE, the individual components of MACE (CV death, nonfatal MI, or nonfatal stroke), hospitalization for unstable angina or heart failure, and revascularization. Additional analyses were performed by gender and age to investigate changes from baseline in HbA_1c_ and body weight, adverse events (AEs), and hypoglycemia, as defined by the American Diabetes Association (ADA) [17].

For comparison of outcomes between men and women, estimated hazard ratios (HRs) and associated confidence intervals (CIs) were determined by a Cox proportional hazards model with an interaction between treatment (semaglutide, placebo) and gender as a fixed factor. Efficacy and safety were assessed by age group using post hoc subgroup analyses of subjects ≤ 65 and > 65 years. Post-baseline responses for time to first occurrence of MACE, CV death, nonfatal MI, nonfatal stroke, hospitalization for unstable angina or heart failure, revascularization, and change from baseline in HbA_1c_ and body weight were analyzed using a mixed model for repeated measurements with interaction between subgroup, randomized treatment, and baseline value as covariate. No adjustment for multiplicity was performed. A significance level for interaction of 5% was considered significant. To investigate more general linear and non-linear effects of age at baseline, individual outcomes and AEs were modelled as a function of age, controlling for randomized treatment and CVD at baseline via negative-binomial log regression (see “Post hoc analysis by age”). Analyses were based on pooled data using semaglutide 0.5 mg and 1.0 mg doses for MACE and its components, hospitalization due to angina or heart failure, revascularization, and AEs. HbA_1c_ and body weight were reported separately for both semaglutide doses following the statistical methods used in the primary SUSTAIN 6 trial [12].

#### CV risk profile subgroups

To assess the effect of baseline CV risk profiles on outcomes, two separate subgroup analyses were performed: (1) for subjects who had experienced a prior MI or stroke compared with those who had not, and (2) for those with established CVD, defined as prior stroke, ischemic heart disease (including MI), peripheral arterial disease, ≥ 50% arterial stenosis in any artery, coronary revascularization (percutaneous coronary intervention or coronary artery bypass graft), or heart failure vs those with CV risk factors alone and no manifestations of CVD as defined above. The risk classification in the latter subgroup comparison differs from the prespecified group of evidence of CVD in SUSTAIN 6, in which subjects with CKD stage 3 or higher were included [12]. In this post hoc analysis, however, subjects with CKD were included in the CV risk factor alone group to reflect the usual definition of established CVD used in clinical practice. Statistical analyses were carried out using Cox proportional hazards models for time to first MACE with treatment, and treatment by subgroup interaction, if applicable, as fixed factor(s). No adjustment for multiplicity was performed. A significance level for interaction of 5% was considered significant.

## Results

### Post hoc analysis by gender

**Fig. 1:**
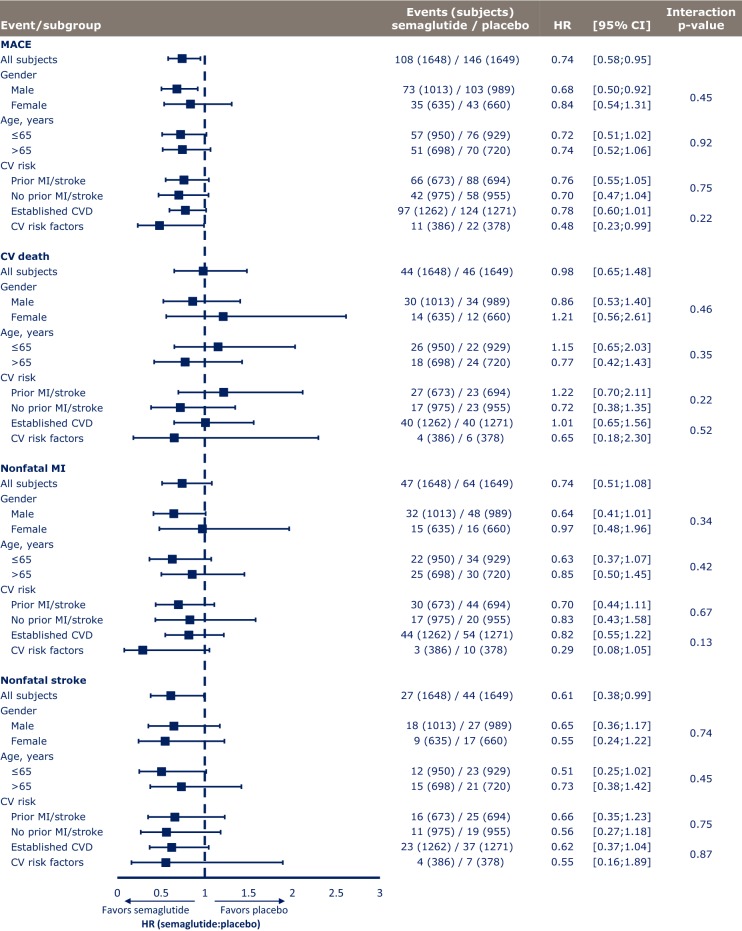
Treatment differences in MACE and MACE components in SUSTAIN 6. Analysis of time from randomization to first event adjudication committee-confirmed event. Subjects were censored at their planned end-of-trial visit, last direct subject-site contact or all-cause death of the subject, whichever occurred first. Estimated HRs and associated CIs are from a Cox proportional hazards model with an interaction between treatment (semaglutide, placebo) and subgroups as fixed factors. The p-values are 2-sided for test for heterogeneity of treatment between subgroups. *CI* confidence interval, *CV* cardiovascular, *CVD* cardiovascular disease, *HR* hazard ratio, *MI* myocardial infarction

**Fig. 2:**
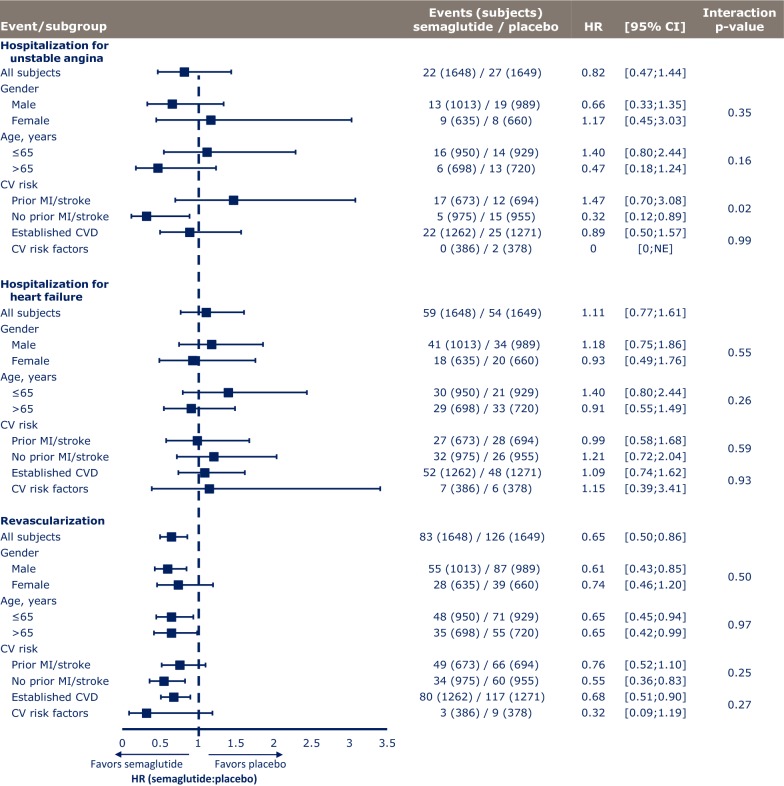
Treatment differences in hospitalization for unstable angina or heart failure, and revascularization in SUSTAIN 6. Analysis of time from randomization to first event adjudication committee-confirmed event. Subjects were censored at their planned end-of-trial visit, last direct subject-site contact or all-cause death of the subject, whichever occurred first. Estimated HRs and associated CIs are from a Cox proportional hazards model with an interaction between treatment (semaglutide, placebo) and subgroups as fixed factors. The p-values are 2-sided for test for heterogeneity of treatment between subgroups. *CI* confidence interval, *CV* cardiovascular, *HR* hazard ratio, *NE* non-estimable

Among 3297 subjects in the SUSTAIN 6 study population, 2002 were male and 1295 were female (Table 1A). There were no clear differences in subject demographics or key baseline characteristics between men and women, with the exception of weight (men tended to be heavier) and smoking status (51.7 vs 26.1% and 56.1 vs 23.0% of men vs women had a history of smoking in the semaglutide and placebo groups, respectively). Similar proportions of male and female subjects completed the trial and treatment. MACE occurred in lower proportions of subjects treated with semaglutide vs placebo in both men and women, and this overall benefit was independent of gender (p = 0.45 for interaction) (Fig. 1). The same pattern was noted across the individual MACE components of CV death, nonfatal MI and nonfatal stroke; lower or similar proportions of both men and women experienced events with semaglutide vs placebo, and p-values for interaction were nonsignificant (p = 0.46, p = 0.34 and p = 0.74, respectively, for each endpoint) (Fig. 1). Gender had no apparent effect on first hospitalization for unstable angina (p = 0.35 for interaction) or heart failure (p = 0.55 for interaction), or time to first revascularization procedure (p = 0.50 for interaction; Fig. 2).

**Table 1:** Baseline characteristics and demographics of subjects in the SUSTAIN 6 trial

A. Post hoc analysis by gender				
	Semaglutide		Placebo	
	Male	Female	Male	Female
Subject demographics				
Full analysis set, N	1013	635	989	660
Trial completers, n (%)	959 (94.7)	602 (94.8)	926 (93.6)	623 (94.4)
Treatment completers, n (%)	773 (76.3)	481 (75.7)	788 (79.7)	514 (77.9)
Baseline characteristics^a^				
Age, years	64.6 (7.3)	64.8 (7.1)	64.6 (7.6)	64.6 (7.5)
Body weight, kg	96.7 (20.5)	85.4 (19.0)	95.8 (21.0)	86.0 (18.4)
BMI, kg/m^2^	32.3 (5.9)	33.7 (6.6)	32.1 (6.0)	33.9 (6.3)
Diabetes duration, years	13.9 (8.1)	14.5 (8.4)	13.5 (8.0)	13.8 (8.1)
HbA_1c_, %	8.6 (1.4)	8.8 (1.6)	8.6 (1.4)	8.8 (1.6)
Smoking status (never/previous/current), %	33.5/51.7/14.8	65.4/26.1/8.5	30.1/56.1/13.7	66.8/23.0/10.2

B. Post hoc analysis by age				
	Semaglutide		Placebo	
	≤ 65 years	> 65 years	≤ 65 years	> 65 years
Subject demographics				
Full analysis set, N	950	698	929	720
Trial completers, n (%)	899 (94.6)	662 (94.8)	875 (94.2)	674 (93.6)
Treatment completers, n (%)	745 (78.4)	509 (72.9)	745 (80.2)	557 (77.4)
Baseline characteristics^b^				
Age, years	59.7 (4.1)	71.4 (4.7)	59.2 (4.3)	71.6 (4.5)
Females, %	38.4	38.7	40.9	38.9
Body weight, kg	94.0 (21.1)	90.0 (19.8)	93.0 (21.4)	90.5 (19.3)
BMI, kg/m^2^	33.3 (6.4)	32.2 (5.9)	33.1 (6.4)	32.4 (5.8)
Diabetes duration, years	12.6 (7.2)	16.4 (8.9)	12.4 (7.4)	15.2 (8.5)
HbA_1c_, %	8.9 (1.6)	8.4 (1.2)	8.9 (1.6)	8.4 (1.3)
Smoking status (never/previous/current), %	47.0/37.1/16.0	44.1/48.4/7.5	45.3/39.0/15.7	44.2/47.9/7.8

C. Post hoc analyses by CV risk profile at baseline								
	Semaglutide		Placebo		Semaglutide		Placebo	
	Prior MI/stroke	No prior MI/stroke	Prior MI/stroke	No prior MI/stroke	Established CVD	CV risk factors	Established CVD	CV risk factors
Full analysis set, N	673	975	694	955	1262	386	1271	378
Baseline characteristics								
Age, years	63.8 (7.5)	65.2 (6.9)	63.6 (7.9)	65.3 (7.2)	64.2 (7.3)	66.1 (6.5)	64.2 (7.7)	66.0 (6.7)
Female, n (%)	208 (30.9)	427 (43.8)	225 (32.4)	435 (45.5)	445 (35.3)	190 (49.2)	463 (36.4)	197 (52.1)
Diabetes duration, years	13.7 (8.5)	14.5 (8.0)	13.3 (8.1)	13.8 (8.0)	14.0 (8.4)	14.8 (7.6)	13.3 (7.9)	14.6 (8.2)
BMI, kg/m^2^	32.6 (6.0)	33.0 (6.4)	32.7 (6.2)	32.8 (6.2)	32.8 (6.1)	32.8 (6.5)	33.0 (6.2)	32.3 (6.1)
HbA_1c_, %	8.8 (1.6)	8.7 (1.4)	8.7 (1.5)	8.7 (1.4)	8.7 (1.5)	8.7 (1.4)	8.7 (1.5)	8.7 (1.5)
CV risk factors								
Systolic blood pressure, mmHg	134.6 (17.7)	136.9 (17.3)	134.9 (17.1)	135.5 (16.6)	135.5 (17.5)	137.5 (17.5)	134.9 (16.6)	136.5 (17.4)
Diastolic blood pressure, mmHg	76.8 (9.9)	77.1 (10.1)	76.7 (10.4)	77.4 (9.8)	76.7 (10.0)	77.9 (9.8)	77.0 (10.2)	77.5 (9.6)
Total cholesterol, mmol/L [mean (CoV)]	4.2 (26.8)	4.4 (25.5)	4.2 (27.9)	4.3 (26.5)	4.3 (26.9)	4.4 (23.6)	4.2 (27.6)	4.4 (25.4)
eGFR, mL/min/1.73 m^2^ [mean (CoV)]	72.1 (39.2)	70.1 (41.4)	73.8 (39.8)	69.0 (44.6)	72.9 (38.7)	64.9 (44.8)	73.9 (39.7)	61.7 (49.2)
Current smoker, n (%)	106 (15.8)	98 (10.1)	109 (15.7)	93 (9.74)	170 (13.5)	34 (8.8)	167 (13.1)	35 (9.3)
Manifestation of CVD								
Prior MI, n (%)	530 (78.8)	–	542 (78.1)	–	530 (42.0)	–	542 (42.6)	–
Ischemic heart disease, n (%)	571 (84.8)	417 (42.8)	589 (84.9)	417 (43.7)	988 (78.3)	–	1006 (79.2)	–
Prior stroke, n (%)	191 (28.4)	–	210 (30.3)	–	191 (15.1)	–	210 (16.5)	–
Peripheral arterial disease, n (%)	87 (12.9)	139 (14.3)	89 (12.8)	138 (14.5)	226 (17.9)	–	227 (17.9)	–
≥ 50% arterial stenosis, n (%)	327 (48.6)	240 (24.6)	361 (52.0)	239 (25.0)	567 (44.9)	–	600 (47.2)	–
Percutaneous coronary intervention, n (%)	327 (48.6)	163 (16.7)	342 (49.3)	180 (18.8)	490 (38.8)	–	522 (41.1)	–
Coronary artery bypass graft, n (%)	182 (27.0)	106 (10.9)	182 (26.2)	107 (11.2)	288 (22.8)	–	289 (227)	–
Heart failure, n (%)	187 (27.8)	194 (19.9)	185 (26.7)	211 (22.1)	381 (30.2)	–	396 (31.2)	–

Data presented as mean (SD) unless otherwise indicated. Data were pooled for semaglutide groups and placebo groups in each SUSTAIN 6 subgroup

*BMI* body mass index, *CoV* coefficient of variation, *CV* cardiovascular, *CVD* cardiovascular disease, *eGFR* estimated glomerular filtration rate, *MI* myocardial infarction, *SD* standard deviation

^a^Numbers are based on an in-trial analysis comprising events with onset on or after the day of randomization and until end of trial

^b^Data were pooled for semaglutide groups and placebo groups in each SUSTAIN 6 subgroup

### Post hoc analysis by age

In the SUSTAIN 6 study, 1879 subjects were 50–65 years of age and 1418 were > 65 years (Table 1B). There were no clear differences in subject demographics or key baseline characteristics between groups. Duration of diabetes was greater in subjects > 65 years compared with those ≤ 65 years (16.4 vs 12.6 years for the semaglutide group and 15.2 vs 12.4 years for the placebo group). Similar proportions of subjects in each age group completed the trial and treatment. HRs for time to first confirmed MACE were all below 1.0 for subjects treated with semaglutide vs placebo, irrespective of age (p = 0.92 for interaction, Fig. 1). Results were consistent for the individual components of MACE across age groups (Fig. 1; p = 0.35, p = 0.42, and p = 0.45 for interaction, respectively, for CV death, nonfatal MI, and nonfatal stroke). Age had no apparent effect on first hospitalization due to unstable angina (p = 0.16 for interaction), heart failure (p = 0.26 for interaction) or revascularization procedures (p = 0.97 for interaction; Fig. 2). A series of regression analyses were conducted to assess more general linear and nonlinear trends for the incidence of MACE by age; since no significant effects were found, these have not been reported further (see Additional file 1).

### Post hoc analysis by CV risk

Pooled key baseline characteristics, CV risk factors, and manifestation of CVD at baseline for the two CV subgroups [subjects with prior MI or stroke vs no prior MI or stroke and subjects with established CVD vs risk factors only (including CKD)] are shown in Table 1C. In total, 1367 subjects had a history of MI or stroke (vs 1930 without prior history) and 2533 had established CVD (vs 764 with CV risk factors only).

**Fig. 3:**
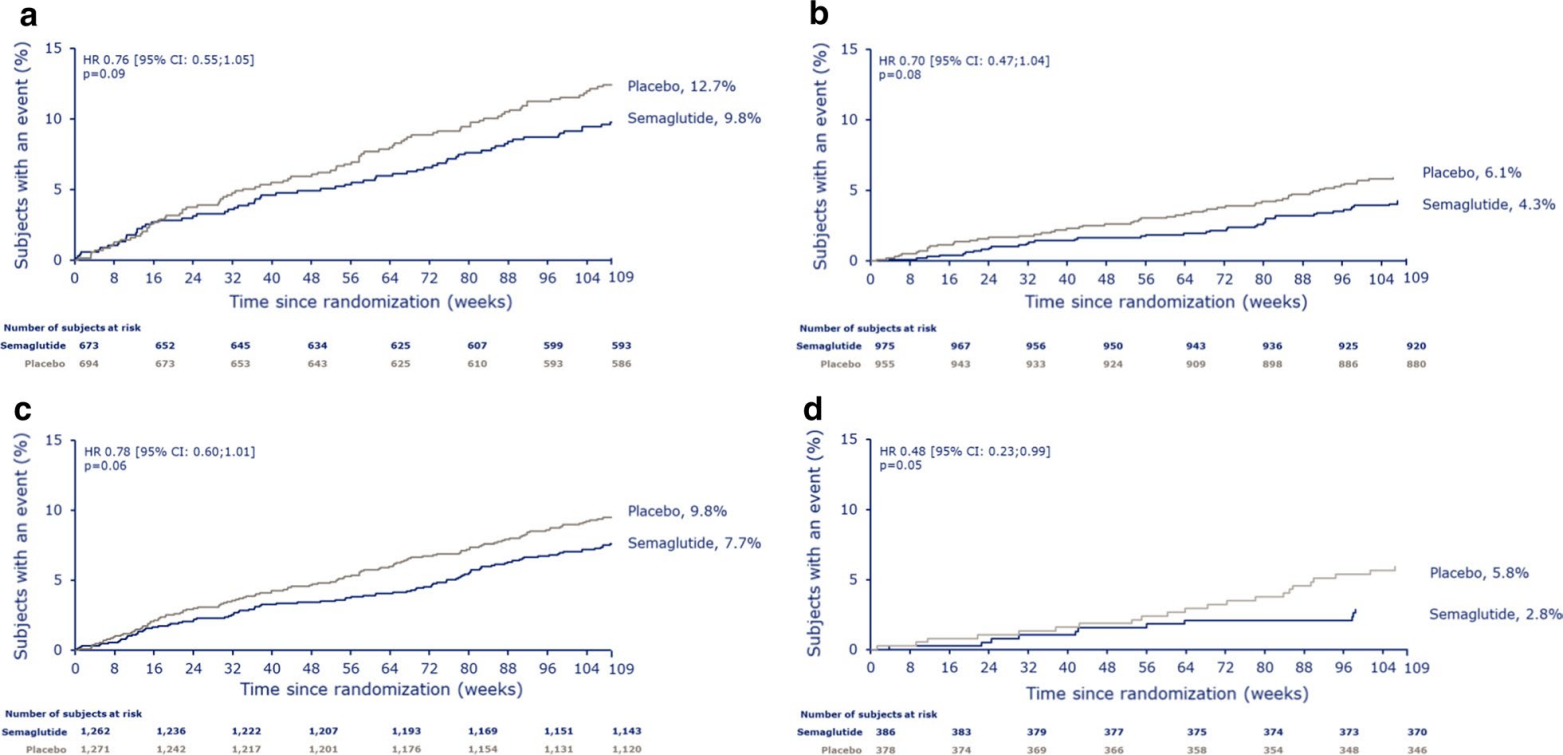
Time from baseline to first confirmed MACE in SUSTAIN 6. In subjects with a prior MI/stroke (**a**) vs no prior MI/stroke (**b**) (p = 0.75 for interaction) and in subjects with established CVD (**c**) vs CV risk factors only (including CKD) (p = 0.22 for interaction) (**d**). Kaplan–Meier estimates: Cox proportional hazards models of time from randomization to first EAC-confirmed MACE in the full analysis set, and treatment by subgroup interaction, if applicable, as fixed factor(s). Data were pooled for semaglutide groups and placebo groups, respectively. *CI* confidence interval, *CKD* chronic kidney disease, *CV* cardiovascular, *CVD* cardiovascular disease, *EAC* event adjudication committee, *HR* hazard ratio, *MACE* major adverse cardiovascular event, *MI* myocardial infarction

Hazard ratios for time to first confirmed MACE were all below 1.0 in subjects treated with semaglutide vs placebo, irrespective of baseline CV risk profile (Fig. 3). Similar results were observed across the individual components of MACE, with the exception of CV death in subjects with a prior MI or stroke or with established CVD (p = 0.22 for interaction between subjects with prior MI or stroke vs no prior MI or stroke; p = 0.52 for interaction between subjects with established CVD vs risk factors only) (Fig. 2).

In the SUSTAIN 6 trial, there was no difference in hospitalization for angina or heart failure between semaglutide and placebo [12], and this result was independent of baseline CV risk profile (Fig. 2). The between-group interaction for unstable angina in subjects with prior MI or stroke compared with no prior MI or stroke was significant (p = 0.02 for interaction), with a significant reduction in hospitalization for unstable angina for semaglutide vs placebo in subjects with no prior MI or stroke (p = 0.03). No significant interactions were noted for hospitalization for heart failure between the various risk groups (Fig. 2; p = 0.59 for interaction between subjects with prior MI or stroke vs no prior MI or stroke and p = 0.93 for interaction between subjects with established CVD vs CV risk factors only).

Semaglutide reduced time to first revascularization vs placebo in the overall study; this result was observed regardless of baseline CV risk profile, with no significant differences between subgroups (p = 0.25 for interaction between subjects with prior MI or stroke vs no prior MI or stroke and p = 0.27 for interaction between subjects with established CVD vs CV risk factors only).

### Reductions in HbA_1c_ and body weight by gender and age

**Fig. 4:**
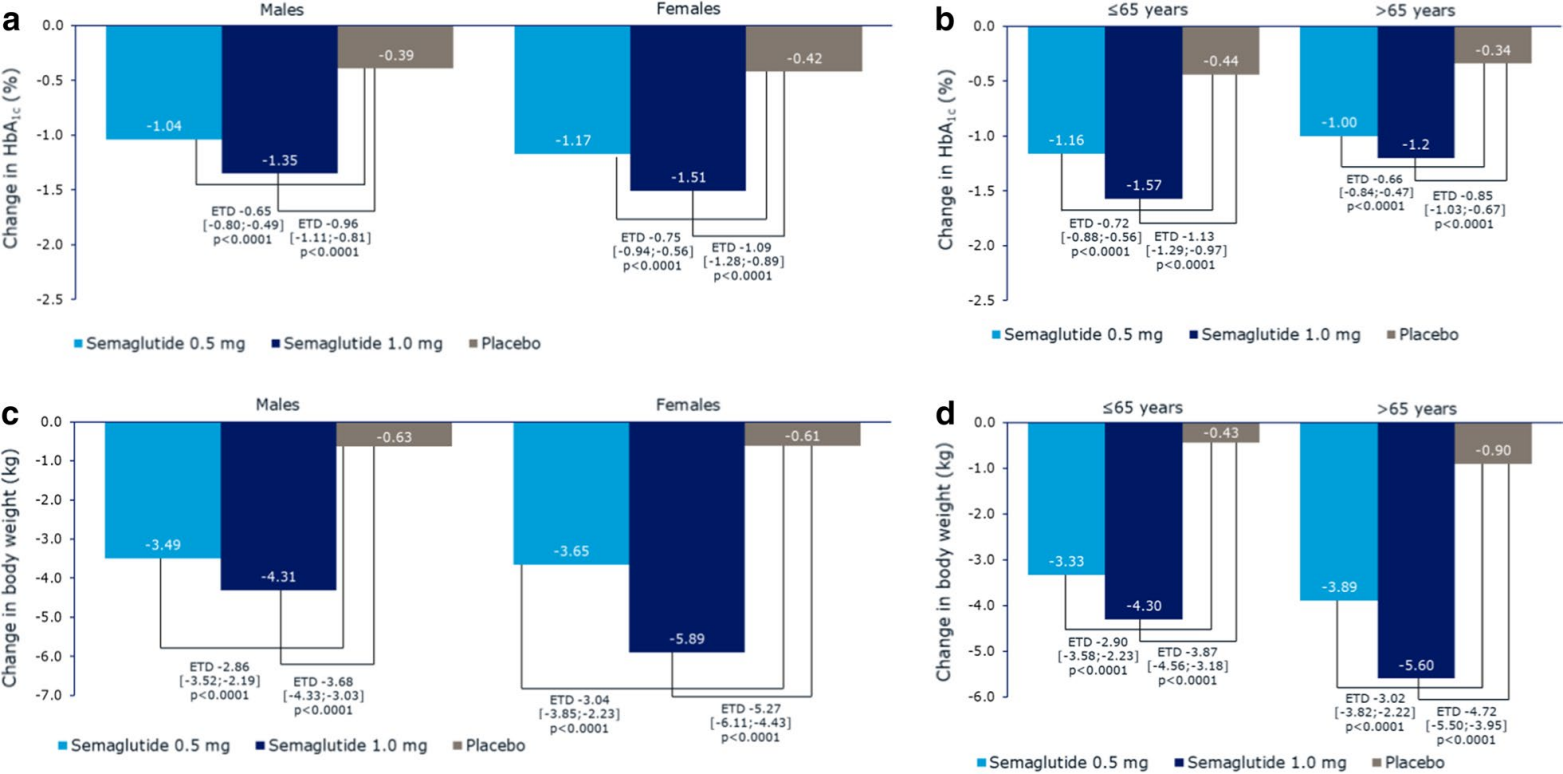
Change from baseline in HbA_1c_ and body weight by gender (**a**, **c**) and age (**b**, **d**) in SUSTAIN 6. *ETD* estimated treatment difference

Significantly greater reductions in HbA_1c_ were achieved by subjects treated with semaglutide than by those receiving placebo, and this decrease was consistent for both genders (ETD for semaglutide 0.5 and 1.0 mg vs placebo: − 0.65% [95% CI − 0.80; − 0.49] and − 0.96% [95% CI − 1.11; − 0.81], respectively in males and − 0.75% [95% CI − 0.94; − 0.56] and − 1.09% [95% CI − 1.28; − 0.89], respectively in females; Fig. 4a) and across age groups (ETDs for semaglutide 0.5 and 1.0 mg vs placebo were − 0.72% [95% CI − 0.88; − 0.56] and − 1.13% [95% CI − 1.29; − 0.97], respectively, in ≤ 65 years, and − 0.66% [95% CI − 0.84; − 0.47] and − 0.85% [95% CI − 1.03; − 0.67], respectively, in > 65 years; Fig. 4b). The p-values for all comparisons between gender and age groups were nonsignificant.

Greater weight loss was observed with semaglutide 0.5 and 1.0 mg compared with placebo in both genders (ETD − 2.86 kg [95% CI − 3.52; − 2.19] and − 3.68 kg [95% CI − 4.33; − 3.03] in males and − 3.04 kg [95% CI − 3.85; − 2.23] and − 5.27 kg [95% CI − 6.11; − 4.43] in females; Fig. 4c) and in subjects ≤ 65 and > 65 years of age (ETDs for semaglutide 0.5 and 1.0 mg vs placebo: − 2.90 kg [95% CI − 3.58; − 2.23] and − 3.87 kg [− 4.56; − 3.18], respectively, and − 3.02 kg [− 3.82; − 2.22] and − 4.72 kg [− 5.50; − 3.95], respectively; Fig. 4d). The p-values for all between-group comparisons were nonsignificant.

### Adverse effects by gender and age

**Fig. 5:**
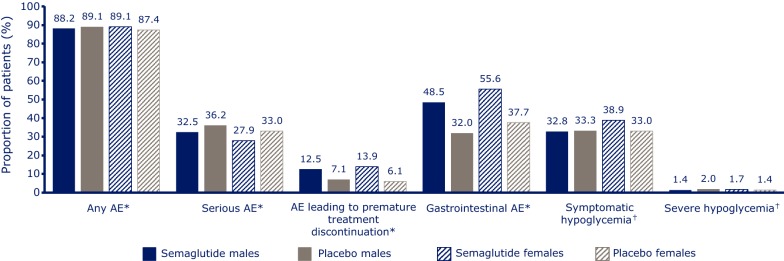
Adverse events by gender in SUSTAIN 6. *On-treatment analysis comprising events with onset from the date of first dose to either the end-of-treatment follow-up visit, the date of last dose plus 42 days, the end-of-trial follow-up visit, or the date of withdrawal from trial, whichever came first (semaglutide males: n = 1007; semaglutide females: n = 635; placebo males: n = 987; placebo females: n = 657). ^†^Full analysis set documented symptomatic hypoglycemia and severe hypoglycemia as defined by the American Diabetes Association [17] (semaglutide males: n = 1013; semaglutide females: n = 635; placebo males: n = 989; placebo females: n = 660). *AE* adverse event

Similar proportions of men and women reported AEs across treatment groups (Fig. 5). Proportions of serious AEs were comparable between semaglutide and placebo for males (32.5 vs 36.2%) and females (27.9 vs 33.0%). The most frequently reported AEs were gastrointestinal (GI) in nature, and they occurred more often with semaglutide than placebo in both males and females. Female subjects reported more GI AEs in all groups compared with men (55.6 vs 48.5% for semaglutide and 37.7 vs 32.0% for placebo). Comparable proportions of men and women prematurely discontinued treatment due to AEs (12.5 and 13.9%, respectively). A similar proportion of males and females reported hypoglycemia (symptomatic as well as severe) with semaglutide as well as placebo treatment.

To investigate the potential effects of age on AEs, a series of regression analyses were conducted. One in-trial AE type was chosen (GI events) and tested both for treatment-dependent and treatment-independent linear and non-linear effects of age on AE incidence; no clear or consistent patterns or significant effects were found (see Additional file 1).

## Discussion

Treatment guidelines recommend the use of antidiabetes agents with proven CV benefits as second-line therapy in T2D populations with CVD [2, 4, 18]. All currently approved injectable GLP-1RAs have demonstrated CV safety (non-inferiority) in CVOTs [12, 19–23], but only four have demonstrated both CV safety and superiority relative to standard of care [12, 20, 22, 23]. While there appears to be a class effect among human-based GLP-1 analogs with respect to CV benefit, additional studies are needed to further elucidate whether CV protective effects are provided by all GLP-1RAs [24, 25].

Semaglutide has demonstrated a consistent effect with respect to glycemic efficacy and safety in T2D populations across the spectrum of care [7–14, 26–28]. In SUSTAIN 6, subjects with T2D at high CV risk treated with semaglutide had a 26% lower risk of the primary composite outcome of first MACE (death from CV causes, nonfatal MI, or nonfatal stroke) vs those receiving placebo over 2 years (p = 0.02 for superiority [post hoc]) [12]. Results of this post hoc analysis of the SUSTAIN 6 trial suggest the beneficial effects of semaglutide vs placebo were similar for men and women, for both younger (50–65 years) and older (> 65) populations studied, and for subjects with varying CV risk profiles at baseline. Similar results were observed with another once-weekly GLP-1RA (exenatide), in which subgroup analyses showed a consistent reduction in MACE across a range of baseline characteristics, including gender, age, and previous CV events [29]. Gender is an important determinant of CV risk within T2D [30]. Historically, women have reached target values for modifiable CV risk factors, including HbA_1c_, systolic and diastolic blood pressure, and lipid levels, less frequently than men [31–33]. In the present post hoc analysis, the decreased risk of first occurrence of MACE associated with semaglutide treatment vs placebo demonstrated in SUSTAIN 6 [12] was comparable for both genders, and overall event rates were similar or lower for women than men. Given the scarcity of available evidence for the effects of T2D treatment on CV outcomes in women and the increased emphasis on CV risk reduction [2], additional analysis of CV outcome trials in T2D by gender could help individualize optimal care and risk management for all patients with a history of or risk factors for CVD.

Cardiovascular risk reduction in populations > 65 years deserves considerable attention alongside the efficacy and safety associated with any intervention. However, trials specifically examining the effect of antihyperglycemic treatment on CV events in older populations are lacking. In this analysis, treatment with semaglutide reduced the risk of a MACE and its components in subjects aged 50‒65 and > 65 years. Further subgroup analysis in subjects > 75 years could not be conducted due to insufficient numbers for accurate assessment. A post hoc analysis from the LEADER (Liraglutide Effect and Action in Diabetes: Evaluation of Cardiovascular Outcome Results) trial examined the effects of liraglutide vs placebo in subjects aged ≥ 75 years (n = 836) and in those aged 60–74 years (n = 6183) [34]. Treatment with liraglutide led to a greater MACE risk reduction in subjects aged ≥ 75 years (HR: 0.66 [95% CI 0.49; 0.89]) compared with those aged 60‒74 years (HR: 0.95; [95% CI 0.83; 1.09]) (p = 0.05 for interaction).

A pre-existent history of MI or stroke is an important CV risk determinant in subjects with T2D [35, 36]. Therefore, the present post hoc analysis divided subjects based on clinically relevant factors (prior MI or stroke vs no prior MI or stroke and established CVD vs risk factors alone) to allow for exploration of CV outcomes across these CV risk subgroups. Semaglutide demonstrated a consistent reduction in MACE and its components vs placebo, regardless of baseline CV risk profile. These results may be compared with those of a post hoc analysis of the LEADER trial, which demonstrated similar incidence rates of MACE in the liraglutide vs placebo group in subjects with a relatively low baseline CV risk (defined as isolated CKD, hypertension with left ventricular hypertrophy, New York Heart Association Class II or III heart failure, and left ventricular systolic or diastolic dysfunction) [37]. Given the low number of subjects and CV events for subjects without established CVD in both analyses, however, these results should be interpreted with caution. The REWIND (Researching Cardiovascular Events with a Weekly Incretin in Diabetes) trial assessed the effect of dulaglutide, a GLP-1RA, vs placebo added to standard of care in 9901 subjects with T2D. In this trial, the majority of subjects did not have established CVD at baseline, and the highly anticipated results from the trial will add to the body of evidence on the potential role for GLP-1RAs on CV risk reduction in a broad range of individuals with T2D [38].

The current analysis evaluated the tolerability of semaglutide across genders and both age groups (50‒65 and > 65 years). Overall, a higher proportion of women reported GI AEs compared with men, although the rate difference between semaglutide and placebo groups was similar for both. Of note, the rate of premature treatment discontinuation due to AEs was comparable between genders, suggesting that the higher reported occurrence of GI AEs in women did not result in increased drug discontinuation. While it is necessary to examine the safety of semaglutide in older populations with T2D, especially in those at risk for retinopathy or kidney complications, these points are not discussed in this analysis and will be explored in future trials [39, 40].

Limitations of the current analysis include the relatively short duration of follow-up (2.1 years) and the relatively small number of MACE, both in the semaglutide group (108/1648 subjects; 6.6%) and in subjects randomized to receive placebo (146/1649 subjects; 8.9%), leading to weaker statistical power in the subgroup results. In addition, the older age group in SUSTAIN 6 included both those with established CVD and those with CV risk factors alone, while the younger group of subjects included only those with established CVD. These differences limit the ability to make assumptions about the consistency of clinical benefit in older T2D populations at high CV risk. However, when CV risk composition of all subjects in these groups was explored in this subgroup analysis, the results suggested a similarity irrespective of age. Finally, considering the nature of subgroup analyses and the risk of false positive effects with a large number of comparisons [41], any conclusions should be interpreted cautiously and in the context of all available data in the literature.

## Conclusion

In this post hoc analysis of the SUSTAIN 6 trial, once-weekly semaglutide vs placebo reduced the risk of MACE in all subjects regardless of gender, age (50‒65 and > 65 years), or baseline CV risk profile (prior MI or stroke vs no prior MI or stroke or established CVD vs CV risk factors alone). In addition, similar reductions in HbA_1c_ and weight were observed with semaglutide compared with placebo across gender and age groups, and safety profiles were comparable between men and women and in subjects above or below the age of 65 years.

## Additional file

**Additional file 1.** Age regression analyses.

## Data Availability

The subject level analysis data sets for the research presented in the publication are available from the corresponding author on reasonable request.

## Abbreviations

AE: adverse events
ADA: American Diabetes Association
CI: confidence interval
CKD: chronic kidney disease
CV: cardiovascular
CVD: cardiovascular disease
ETD: estimated treatment difference
GLP-1RA: glucagon-like peptide-1 receptor agonist
HR: hazard ratio
MACE: major adverse cardiac event
MI: myocardial infarction
T2D: type 2 diabetes
