# Supplementary material for: Cardiovascular risk reduction with once-weekly semaglutide in subjects with type 2 diabetes: a post hoc analysis of gender, age, and baseline CV risk profile in the SUSTAIN 6 trial
Source: Cardiovasc Diabetol. 2019 Jun 6;18:73. doi: 10.1186/s12933-019-0871-8 (PMC6551895; doi:10.1186/s12933-019-0871-8)
Supplement: Supplementary file 1 — Additional file 1. Age regression analyses. [file 12933_2019_871_MOESM1_ESM.docx]

**ADDITIONAL FILE**

A series of regression analyses were performed to investigate the effect of age at baseline on the risks for major adverse cardiac events (MACE) and gastrointestinal adverse events (GI AEs). Operating under the assumption that the event rate (number of events per exposure time) followed a negative-binomial distribution, both treatment-dependent and treatment-independent non-linear (spline) and linear effects of age at baseline were tested while controlling for randomized treatment and cardiovascular disease status at baseline) for MACE and GI AEs. None of the regression analyses showed any effect of age at baseline on the incidence risk for either endpoint (MACE or GI AEs; p>0.1) (Table).

| Statistical test for effect of age on endpoint | p | |
| --- | --- | --- |
|  | MACE | GI AEs |
| Treatment-dependent non-linear vs treatment-independent non-linear | 0.67 | 0.91 |
| Treatment-dependent non-linear vs treatment-dependent linear | 0.59 | 0.78 |
| Treatment-independent non-linear vs treatment-independent linear | 0.51 | 0.51 |
| Treatment-independent linear vs no effect of age | 0.41 | 0.14 |

AE, adverse event; GI, gastrointestinal; MACE, major adverse cardiac event.
